# Fusion of Multi-Task fMRI Data: Guided Solutions for IVA and Transposed IVA

**DOI:** 10.3390/s26020716

**Published:** 2026-01-21

**Authors:** Emin Erdem Kumbasar, Hanlu Yang, Vince D. Calhoun, Tülay Adalı

**Affiliations:** 1 Department of CSEE, University of Maryland Baltimore County, Baltimore, MD 21250, USA; hyang3@umbc.edu (H.Y.); adali@umbc.edu (T.A.); 2 Tri-Institutional Center for Translational Research in Neuroimaging and Data Science (TReNDS), Georgia State University, Atlanta, GA 30303, USA; vcalhoun@gatech.edu; 3 Georgia Institute of Technology, Emory University, Atlanta, GA 30332, USA

**Keywords:** independent vector analysis (IVA), constrained IVA, transposed IVA, fMRI, sMRI, data fusion, behavioral variables

## Abstract

Independent vector analysis (IVA) has emerged as a powerful tool for fusing and analyzing functional magnetic resonance imaging (fMRI) data. Applying IVA to multi-task fMRI data enhances analytical power by capturing the relationships across different tasks in order to discover their underlying multivariate relationship to one another. Incorporation of prior information into IVA enhances the separability and interpretability of estimated components. In this paper, we demonstrate successful fusion of multi-task fMRI feature data under two settings: constrained IVA and constrained transposed IVA (tIVA). We show that using these methods for fusing multi-task fMRI feature data offers novel ways to improve the quality and interpretability of the analysis. While constrained IVA extracts components linked to distinct brain networks, tIVA reverses the roles of spatial components and subject profiles, enabling flexible analysis of behavioral effects. We apply both methods to a multi-task fMRI dataset of 247 subjects. We demonstrate that for task-based fMRI, structural MRI (sMRI) references provide a better match for task data than resting-state fMRI (rs-fMRI) references, and using sMRI priors improves identification of group differences in task-related networks, such as the sensory-motor network during the Auditory Oddball (AOD) task. Additionally, constrained tIVA allows for targeted investigation of the effects of behavioral variables by applying them individually during the analysis. For instance, by using the letter number sequence subtest, a measure of working memory, as a behavioral constraint in tIVA, we observed significant group differences in the auditory and sensory-motor networks during the AOD task. Results show that the use of two constrained approaches, guided by well-aligned structural and behavioral references, enables a more comprehensive analysis of underlying brain function as modulated by task.

## 1. Introduction

Multimodal data fusion has emerged as a powerful approach for analyzing multiple datasets, as it improves the identification of useful features by capturing relationships across datasets [1]. Its impact is particularly notable in the medical field, where combining information from diverse sources can lead to more accurate and insightful results [2]. Functional magnetic resonance imaging (fMRI), a widely used neuroimaging technique, enables investigation of brain activity [3], leveraging the effect dependent on blood oxygenation level (BOLD) [4]. In particular, fusion of multi-task fMRI data offers significant clinical value, particularly in identifying biomarkers associated with psychiatric disorders [5]. To more effectively analyze subject-level differences across tasks, the fusion is performed on task-regressed multi-task fMRI feature data, which provides feature-level representations tailored to each task. To fully leverage the potential of such applications, it is essential to employ data fusion techniques that effectively model and facilitate interactions across datasets [5].

Independent vector analysis (IVA), an extension of independent component analysis (ICA) to multiple datasets, has proven to be highly effective in capturing inter-dataset relationships, making it an attractive method for data fusion [1,6]. As a data-driven technique, IVA requires minimal assumptions about the underlying data, which enhances its flexibility and applicability across diverse domains [7]. Unlike ICA, IVA models both within-dataset and across-dataset dependencies by introducing source component vectors (SCVs), which aligns corresponding sources across datasets and maximizes their statistical dependence [6,8,9]. This formulation enables IVA to fully exploit interactions between datasets. In the context of multi-task fMRI, IVA has been shown to successfully reveal abnormal brain activations by differentiating patterns between schizophrenia patients and healthy controls [5,10,11,12]. An alternative approach, transposed IVA (tIVA), extends this approach by transposing the datasets, thereby interchanging the roles of spatial sources and temporal profiles [13]. This shift in roles allows the separation of task-specific activation profiles rather than spatial sources, enabling a comparative analysis of brain network activations across different tasks for different profiles.

Incorporating prior information is an effective strategy for improving the separability and interpretability of IVA-based solutions [14]. By guiding the decomposition toward known reference patterns, prior information provides valuable insight into the underlying sources and enables more accurate source separation. It can also accelerate algorithmic convergence, thereby reducing overall computational cost [15,16]. These advantages make constrained IVA a promising approach for multi-task fMRI data fusion. However, the performance of this method strongly depends on the quality and relevance of the references that are used.

One key challenge in multi-task fMRI feature analysis is the lack of task-specific reference templates. Although resting-state fMRI (rs-fMRI) data are commonly used as references in constrained IVA, prior studies have shown that they may not optimally correspond to task-related activations [17]. Resting-state data are analyzed in their raw form and, for each subject, are represented as a time-points-by-voxels matrix. In contrast, task-based feature data are obtained by regressing the fMRI signal onto task-related regressors, producing feature-level representations across all voxels—resulting in a single feature vector per subject. As a result, rs-fMRI captures intrinsic, global brain activity, while task-based fMRI features isolate activations specifically associated with task performance, leading to a modality mismatch that can reduce analytical effectiveness. Moreover, recent studies [18,19] have further highlighted that, although rs-fMRI and task-based fMRI share overlapping information, they also exhibit distinct activation patterns.

An alternative reference modality is structural MRI (sMRI), which provides high-resolution anatomical information that directly complements the functional signals captured in task-based fMRI. Because task-based fMRI is modeled to emphasize localized, task-specific activations, sMRI references naturally align with this feature modality by offering anatomically grounded priors. Anatomical priors have been shown to help predict individual variations in task-related activations [20]. This correspondence enables constrained IVA to better capture meaningful functional variability and group differences, ultimately improving source separation and enhancing the interpretability of task-related activation patterns. The sMRI references examined in this study were derived using source-based morphometry (SBM), which applies ICA to a group of gray matter data to identify spatial covarying voxel patterns [21,22]. This approach enables the investigation of structural features. Throughout the remainder of the paper, these SBM-derived structural components are referred to as “sMRI references”.

In the tIVA framework for multi-task fMRI analysis, the change in data dimensionality leads to a “sample-poor regime”. Unlike the standard IVA setting, where the analysis is performed over a large number of samples (voxels), tIVA operates with a much smaller sample size (subjects), which can make the estimation less stable. Therefore, tIVA has not been viewed as an attractive solution for fusing multi-task fMRI feature data. However, with the incorporation of prior information, the stability of the estimations can be substantially improved, making the use of constraints even more valuable in this framework. Informative constraints help compensate for the reduced sample size inherent in this setting. In multi-task fMRI analysis, constrained tIVA enables the analysis of brain activation patterns in relation to behavioral variables. Unlike standard constrained IVA, which applies constraints to the sources, constrained tIVA imposes constraints on the profiles. By incorporating behavioral measures as references, this framework allows the identification of task-related activation patterns linked to behavioral patterns, thereby providing insights into how behavior modulates brain activity across tasks. Establishing relationships between spatial components and behavioral or clinical variables holds significant value for biomarker discovery in neurological and psychiatric conditions, as it aids in the interpretation of estimated components [23,24,25]. Moreover, uncovering associations between components and behavioral measures can help delineate disease subtypes and subject subgroups, which is crucial for advancing personalized treatment strategies [24,26].

In this study, we employ threshold-free constrained IVA (tf-cIVA), introduced in [16], as our constrained IVA framework. Unlike typical constrained approaches, the threshold-free constraint maximizes the similarity between references and estimated sources without requiring a constraint parameter, this design enables greater flexibility, as the post-analysis correlation values between estimated sources and references serve as a more reliable measure of reference quality. In previous works, this algorithm has been applied to the analysis of raw fMRI data. In contrast, the fMRI dataset used here has been regressed to the feature level using task-specific response models. To our knowledge, this framework has not previously been applied to multi-task fMRI feature analysis and only constant constraint parameters were used in preliminary work [17]. Moreover, within the tIVA framework, the incorporation of constraints has not been explored, making this a new application of the method for multi-task fMRI feature data.

For the fusion of multi-task fMRI feature data, we introduce two novel applications of flexible constrained approaches. Within the constrained IVA framework, tf-cIVA is used on the multi-task fMRI data to evaluate which reference modality provides the best guidance. We compare sMRI references to rs-fMRI references, particularly for detecting group differences. This comparison is evaluated using a dataset of 138 healthy controls and 109 individuals with schizophrenia who completed three tasks: auditory oddball (AOD), Sternberg item recognition paradigm (SIRP), and sensory motor (SM) [27]. Our results show that with sMRI references, the constrained component associated with the sensory-motor network exhibited significantly higher activation in healthy controls compared with schizophrenia patients for both the AOD and SM tasks, an effect not captured with rs-fMRI references. For the SM task, activations within the default mode network were also significant with sMRI references but not with rs-fMRI references. In addition, correlation values with the references were consistently higher when using sMRI, supporting its stronger alignment with multi-task fMRI. These findings are consistent with prior clinical research, as abnormalities in these networks have been observed in schizophrenia patients. Overall, tf-cIVA with sMRI references provides a more effective framework for multi-task fMRI analysis than with rs-fMRI references.

In the constrained tIVA setting, we fuse this dataset to investigate the relationships between brain activation patterns and behavioral variables. Cognitive test scores from four well-established assessments, Wechsler Adult Intelligence Scale-Third Edition (WAIS-III) [28], Wechsler Memory Scale-Third Edition (WMS-III) [29], and Hopkins Verbal Learning Test–Revised (HVLT) [30] serve as reference measures. These assessments are widely recognized for their reliability and their ability to accurately capture cognitive impairments in schizophrenia, evaluating domains such as working memory, visual memory, verbal memory, and learning capacity. Using constrained tIVA, we show that these references effectively reveal group differences in task-related activation patterns associated with these cognitive functions. To account for the interdependence among the cognitive measures, each reference is applied individually, enabling the isolation of task-specific activations and the assessment of their significance across the different fMRI tasks. For the behavioral variables we have used as constraints, we were able to identify corresponding fMRI components showing significant activations in the motor, sensorimotor, auditory, left and right frontoparietal. Previous studies [12,31,32] supported our findings as these regions have been consistently associated with working memory deficits in schizophrenia patients.

## 2. Methods

### 2.1. IVA and tIVA

For *K* observed datasets, each dataset can be represented as a linear mixture of *N* statistically independent sources observed over *V* samples (voxels). To model the analysis on *N* sources, each dataset undergoes dimensionality reduction using principal component analysis (PCA), resulting in datasets of the form $X^{\left[k\right]}\in R^{N\times V}$, where *V* denotes the number of voxels for fMRI data. The generative model of IVA can then be formulated using random vector notation as:

(1) $$x^{\left[k\right]}\left(v\right)=A^{\left[k\right]}s^{\left[k\right]}\left(v\right),1\leq k\leq K,$$

where $A^{\left[k\right]}\in R^{N\times N}$ is an invertible mixing matrix for the *k*th dataset and $s^{\left[k\right]}\left(v\right)=\in R^{N\times V}$ is the source matrix. IVA captures dependencies across datasets through the concept of an SCV, defined by concatenating the *n*th source from all *K* datasets as $s_{n}\left(v\right)={\left[s_{n}^{\left[1\right]}\left(v\right),\ldots ,s_{n}^{\left[K\right]}\left(v\right)\right]}^{⊤}$. The objective of IVA is to identify SCVs that are maximally independent while simultaneously maximizing the dependency among components within each SCV. To achieve this, IVA estimates *K* demixing matrices, $W^{\left[k\right]}\in R^{N\times N}$, such that the *N* estimated sources for the *k*th dataset are given by ${\hat{s}}^{\left[k\right]}\left(v\right)=W^{\left[k\right]}x^{\left[k\right]}\left(v\right)$ and the *n*th estimated SCV at sample index *v* can then be expressed as ${\hat{s}}_{n}\left(v\right)={\left[{\hat{s}}_{n}^{\left[1\right]}\left(v\right),\ldots ,{\hat{s}}_{n}^{\left[K\right]}\left(v\right)\right]}^{⊤}\in R^{K}$. Assuming the samples are independently and identically distributed, the IVA cost function can be derived based on the maximum likelihood principle [6] as follows:

(2) $$J_{\mathrm{IVA}}\left(W,\Sigma \right)=-\frac{1}{V}\sum_{v,n}\log p_{n}\left({\hat{s}}_{n}\left(v\right)|\Sigma_{n}\right)-\sum_{k}\log|\det W^{\left[k\right]}|,$$

Here, $p_{n}$ denotes the probability density function (pdf) of the *n*th SCV, and $\Sigma_{n}\in R^{K\times K}$ represents its covariance matrix. The samples are assumed to be independently and identically distributed. By adopting different models for $p_{n}$, various forms of IVA can be defined. For example, a commonly used variant, IVA-G, models the estimated SCV, ${\hat{s}}_{n}$, as following a multivariate Gaussian distribution. In this paper, this IVA-G model is used for constrained IVA. IVA-G relies only on second-order statistics (SOS). Although Laplacian models have been reported to better capture certain non-Gaussian characteristics of brain fMRI data, incorporating prior information into the solution effectively introduces additional information that capture some higher-order statistical structure, thereby reducing practical differences between Gaussian and Laplacian based IVA algorithms. In the tIVA setting, cross-joint-ISI comparisons between Gaussian and Laplacian models showed that the Gaussian shape parameter yielded more stable solutions. Therefore, IVA-G was selected for both IVA and tIVA analyses.

With tIVA, the transpose of the dataset is used so that the original IVA equation for the set of observations $X^{\left[k\right]}$ can be written as,

(3) $${X^{\left[k\right]}}^{⊺}={\left(A^{\left[k\right]}S^{\left[k\right]}\right)}^{⊺}={S^{\left[k\right]}}^{⊺}{A^{\left[k\right]}}^{⊺}$$

**Figure 1:**
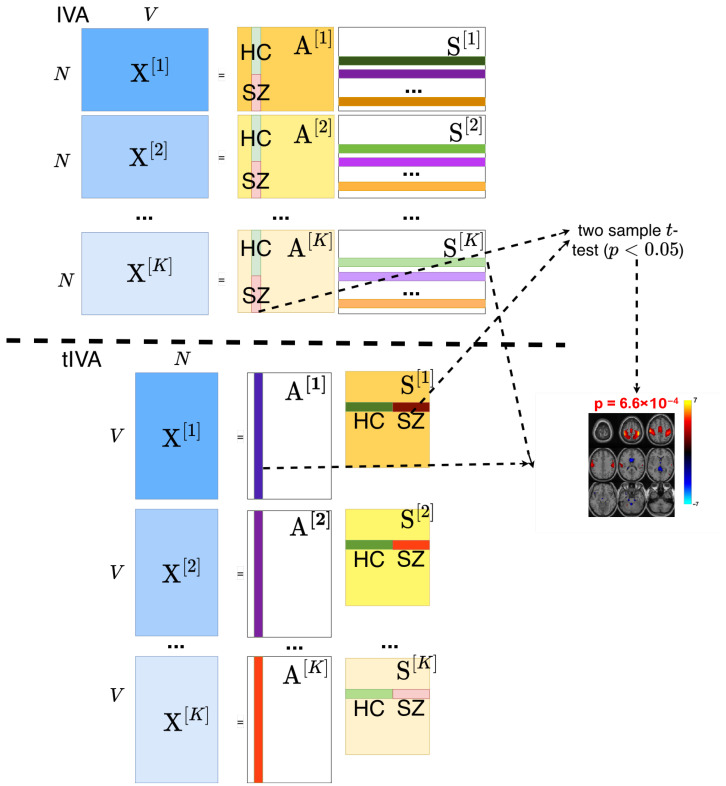
Illustration of changes in roles of profiles and components for IVA and tIVA. Here, $X^{\left[k\right]}$ is task regressed fMRI data of *N* subjects. A two-sample *t*-test is performed on the columns of $A^{\left[k\right]}$ (equivalently, on the rows of $S^{\left[k\right]}$ in tIVA) to assess group differences between healthy controls and schizophrenia patients for the corresponding spatial components. The *p*-value is shown above the example fMRI component on the right. Rows of $S^{\left[k\right]}$ (equivalently, columns of $A^{\left[k\right]}$) represent the *N* spatial components, which correspond to brain activation patterns in the example plot.

In the tIVA framework, the SCVs are constructed from the columns of A, which correspond to the temporal profiles of the sources represented by the rows of S. Effectively, the rows of S in the original IVA formulation now occupy the columns of the mixing matrix, A. This structural change alters the properties of the analysis: whereas in standard IVA the components are independent within each dataset and maximally dependent across datasets, in tIVA these properties now apply to the profiles, i.e., the subject covariations [13]. Consequently, each SCV in tIVA represents a profile across datasets, rather than a set of components from different datasets as in the original IVA model. This shift in the roles of profiles and spatial components is illustrated in the model schematics shown in Figure 1.

### 2.2. Integrating Prior Information

Incorporating prior information into the IVA model can enhance source separation by guiding the decomposition to align with a given set of reference signals. This guidance also facilitates the separation process, leading to reduced computational cost. In our flexible framework, for a set of reference signals $\left\{r_{1},\ldots ,r_{M}\right\}$, the number of references may be less than or equal to the number of sources, i.e., M≤N. To impose the constraint, we adopt the tf-cIVA framework introduced in [16], which does not require any threshold parameters. This approach maximizes the similarity between each reference $r_{n}$ and its corresponding estimated source component while simultaneously promoting dissimilarity between $r_{n}$ and all other estimated components [16]. This process is done with the addition of a regularization term,

(4) $$J_{\mathrm{ref}}\left(W\right)=\sum_{n=1}^{M}\sum_{k=1}^{K}\left(\sum_{\begin{array}{c} m=1\\ m\neq n \end{array}}^{K}\epsilon^{2}\left(r_{n}{\hat{s}}_{m}^{\left[k\right]}\right)-\epsilon^{2}(r_{n},{\hat{s}}_{n}^{\left[k\right]})\right).$$

Using this term, the objective function is reformulated by adding it to the original cost function,

(5) $$L_{\lambda }\left(W,\Sigma \right)=J_{\mathrm{IVA}}\left(W,\Sigma \right)+\frac{\lambda }{2}J_{\mathrm{ref}}\left(W\right)$$

where λ>0 is the regularization parameter for the algorithm.

Since the dissimilarity between each reference and non-corresponding source components is enforced, using references that are not highly correlated with one another helps improve the quality of source separation. In the case of tIVA, the estimated components correspond to profiles. Constrained tIVA follows the same framework as constrained IVA; however, due to the change in decomposition, the profiles are now estimated while maximizing independence across other profiles. Therefore, it is important to note that many behavioral measures used to constrain profiles are not fully independent, often reflecting overlapping or similar constructs. To address this issue in constrained tIVA, the references are not applied simultaneously. Instead, each reference is introduced individually across separate runs. This approach allows for a more precise estimation of the activations associated with each reference and provides a more reliable assessment of the significance of individual behavioral variables without interference from other references.

## 3. Results

For both constrained IVA and constrained tIVA, threshold free constrained IVA-G (tf-cIVA-G) algorithm was employed. In a preliminary analysis of this multi-task fMRI dataset, fixed constraint parameters were used [17]. Following both constrained IVA and tIVA, two-sample *t*-tests were performed on the profiles to evaluate the significance of each component. The resulting *p*-values were corrected using the false discovery rate (FDR) method [33]; components were deemed significant if the corrected values satisfied p<0.05.

In addition to the two-sample *t*-test analysis, effect sizes were calculated using the estimated profile information from the two groups. Cohen’s d was used to quantify the magnitude of the difference between groups, independent of sample size [34]. Reporting effect sizes provides complementary information to *p*-values and is particularly informative in the tIVA setting, where the number of available samples is more limited.

The regularization parameter was set to λ=100, following [16], which provides a good balance between the two terms. For model order selection, constrained IVA was run with an order of N=45, consistent with prior work showing this to be a good match for the dataset [17]. For constrained tIVA, an order of N=6 was selected based on analysis of the cross-joint inter-symbol interference (cross-joint-ISI) [35], a global distance metric that evaluates consistency across runs. Smaller cross-joint-ISI values indicate greater reproducibility and stability. To identify the most stable solution, 100 runs were performed for each order, and the run with the highest reproducibility was selected. Prior research has demonstrated that, for both matrix and tensor factorization methods, the most reproducible run generally yields the most reliable model match, making it the preferred solution [36].

In addition to stability-based order selection, we compared the statistical significance of the constrained components associated with each behavioral variable across the stable model orders. Among these, order N=6 yielded constrained components with *p*-values most closely matching the intrinsic group-level significance of the corresponding behavioral measures.

In this section, we first describe the dataset utilized in the study along with the set of references incorporated for constrained IVA and tIVA. We then present the results of constrained IVA applied to the multi-task fMRI dataset, highlighting comparisons of the estimated components when sMRI and rs-fMRI references are employed. Finally, constrained tIVA is conducted using different behavioral variables on the same dataset to facilitate clinically more meaningful interpretation of the findings.

### 3.1. fMRI Dataset

The fMRI dataset used in this study was obtained from the Mind Research Network Clinical Imaging Consortium Collection [27]. It includes 247 subjects, comprising 138 healthy controls and 109 individuals diagnosed with schizophrenia. Participants completed the AOD, SIRP, and SM tasks. Imaging features were extracted using the Statistical Parametric Mapping (SPM) toolbox [37]. For each subject, voxel-wise linear regression was performed to derive the imaging features. Regressors were constructed by convolving SPM’s hemodynamic response function (HRF) with the task-specific predictors. Using task-specific regressors for each task, a general linear model (GLM) was applied to the fMRI data to obtain feature level task regressed signals, as in [38]. The resulting feature matrices are represented as $X_{o}^{\left[k\right]}\in R^{T\times V}$, where T=247 corresponds to the number of subjects, *V* = 48,546 to the number of voxels, and k=1,2,3 indexes the three task datasets. Details of each task are described below.

#### 3.1.1. Auditory Oddball Task

In the AOD task, subjects were presented with three types of auditory stimuli: standard, novel, and target [27]. These stimuli were delivered in a pseudorandom order. The standard stimulus, a 1 kHz tone, occurred frequently, whereas the novel and target stimuli were presented less often. Participants were instructed to press a designated button whenever they detected the target stimulus, which had a distinct 1.2 kHz tone [39]. The feature for this task was derived using a regressor corresponding to the target stimulus, modeling the target-related responses.

#### 3.1.2. Sternberg Item Recognition Paradigm Task

SIRP differs from the other tasks as it is the only visual task in the dataset. In this task, subjects were presented with sets of 1, 3, or 5 random digits ranging from 0 to 9. They were instructed to memorize the digits during the first 1.5 s, followed by a 0.5-s blank screen, then a 6-s encoding period, and finally a 38-s probe phase. During the probe phase, subjects were shown random digits and were required to press a button whenever a digit from the original set appeared [27]. The feature data for this task was derived using a regressor corresponding to the probe phase.

#### 3.1.3. Sensory Motor Task

The third task, SM task, also involves auditory stimuli. In this task, subjects were presented with 16 distinct auditory tones played sequentially with increasing frequency. After all 16 tones were presented, their order was rearranged. Participants were instructed to press a button whenever a change in tone was detected [27]. The feature data for this task was generated using a regressor obtained by convolving the entire sequence of increasing and decreasing tone blocks, with the resulting average serving as the input feature.

### 3.2. Reference Data

#### 3.2.1. Neuromark fMRI and sMRI References

For constrained IVA, the constraints are applied to the source components, requiring a set of reference signals to guide the decomposition of the fMRI data. Typically, rs-fMRI data are used as reference signals. However, in this study, the data of interest are task-based fMRI features. Therefore, in addition to rs-fMRI references, sMRI references were also employed and compared in terms of their suitability for model matching.

The rs-fMRI references used in this study were obtained from the Neuromark 1.0 template [40], which includes 53 components spanning seven brain networks: subcortical (SC), auditory (AU), sensorimotor (SM), visual (VI), cognitive control (CC), default mode (DM), and cerebellar (CB) networks. For each network, the components were averaged to create a single reference per network. sMRI references were drawn from the Neuromark 3.0 sMRI template [22], and components corresponding to the same seven brain networks were similarly aggregated to provide a set of references comparable with those from rs-fMRI.

#### 3.2.2. Behavioral Variables

For constrained tIVA, due to the interchange of roles between profiles and source components, the constraints are applied to the profiles rather than the sources. This enables a different approach to constraint-based analysis. By constraining the profiles with behavioral features, cognitive measures collected from the subjects through various tests can be evaluated, allowing the significance of these measures and their associated brain activations to be examined.

In this study, the cognitive scores were obtained from the WAIS-III, WMS-III, and HVLT tests. Working memory was assessed using the Letter-Number Sequencing subtest of WAIS-III. Verbal memory and learning were evaluated HVLT and the Logical Memory subtest of WMS-III. Visual memory was measured using the Face Recognition subtest of WMS-III. Each subtest included multiple metrics for analyzing group differences between healthy controls and patients. For each test, the metric demonstrating the highest variability across groups was selected: total raw score for the Letter-Number Sequencing subtest and HVLT, and recognition total score for the Logical Memory and Face Recognition subtests.

### 3.3. Results and Discussion

#### 3.3.1. Comparison of rs-fMRI vs. sMRI References with Constrained IVA

**Figure 2:**
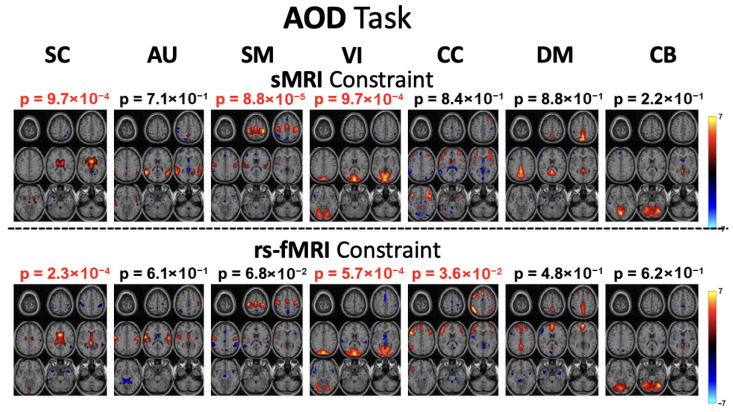
Estimated components for the AOD task using constrained IVA with sMRI and rs-fMRI references. *p*-values observed above the spatial components are calculated with two-sample *t*-test between the profile information of the two groups as described in Figure 1. The lower *p* values indicate a more significant difference for the given component between healthy controls and schizophrenia patients. In the SM network, an active network during AOD task which is known to be associated with schizophrenia, a significant group difference (much lower *p*-value) is observed with sMRI references, whereas this difference is not evident when using rs-fMRI references (p>0.05).

**Figure 3:**
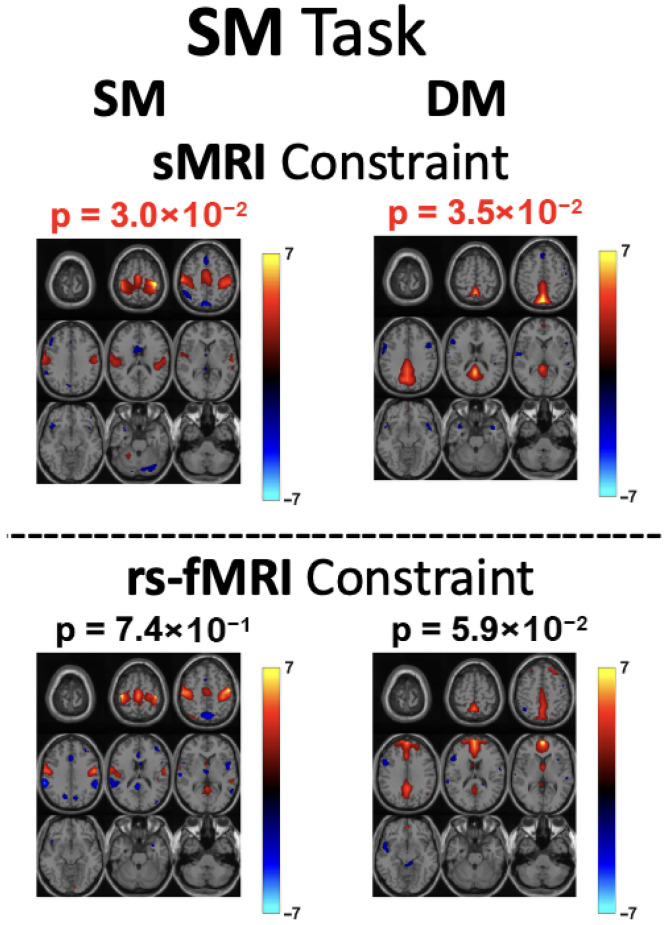
Estimated SM and DM network components for the SM task using constrained IVA with sMRI and rs-fMRI references. Significant activations were observed with sMRI references, whereas no significance was found with rs-fMRI references (p<0.05) for these two networks.

To study the performance with the two sets of chosen references, the analysis focused on the first seven constrained components. Both runs used the same model order (45) and were processed with the tf-cIVA-G algorithm. The evaluation assessed the quality of the separated components, their correlations with the respective references, and the significance of group differences among these seven components under each task. Previous studies [12,17] have reported that, for the AOD and SM tasks, the SM network is among the networks revealing most pronounced group differences. As shown in Figure 2 and Figure 3, for both tasks, the SM network component estimated using rs-fMRI references does not reveal a significant difference between groups. In contrast, when sMRI references are used, a significant group difference in the SM network emerges. Moreover, the estimated components exhibit higher correlations with their corresponding sMRI references across all three tasks. These findings align with prior evidence showing that schizophrenia patients exhibit reduced connectivity within the SM network compared with healthy controls [41,42]. Therefore, detecting a significant group difference in the SM network is both expected and essential, especially for the AOD and SM tasks, which strongly engage this network. Another network showing changes in the significance of group differences under the SM task is the DM network. Prior studies [43,44,45,46] have demonstrated that activations within the DM network differ between schizophrenia patients and healthy controls. Furthermore, it has been shown that this network can be activated during SM tasks [12]. The emergence of significant activation when using sMRI constraints therefore suggests that sMRI references provide a better modality match than rs-fMRI references for modeling task-related activity.

Overall, the correlation values in Table 1 further support that sMRI references provide a better match for multi-task fMRI data. In five of the seven constrained networks, the estimated components show higher correlations with the references across all three tasks compared with those obtained using rs-fMRI references.

For the SIRP task, none of the seven constrained components exhibited significant group differences. However, a significant component showing activation within the visual (VI) network was identified among the unconstrained components for both sets of references. This observation is consistent with previous findings [17], which suggest that leaving some components unconstrained allows the model to recover meaningful patterns not necessarily aligned with the reference data.

Recent studies have demonstrated that structural imaging carries information predictive of individual variations in task-based activations [20]. Moreover, using anatomical information as a prior for task fMRI analysis has proven effective in enhancing model performance [47]. The differences between task-based and resting-state fMRI activations have also been explored in [18], which reported distinct activation patterns between the two modalities. These findings reinforce our argument that rs-fMRI references are not ideally suited for constraining task feature data. Additionally, the task fMRI data in this study were regressed for each individual task to form feature representations, whereas rs-fMRI references are derived from temporal analyses, which introduces a modality mismatch that further limits their suitability for this application.

Averaging components belonging to the same network have enabled network level interpretation and a direct comparison of rs-fMRI and sMRI references. This approach is particularly suitable when multiple components within a network exhibit task-related activations, as it captures the overall spatial representation of the network rather than emphasizing a single component. However, averaging may potentially blur the spatial specificity and result in smoother sMRI maps. Future studies may further investigate component level matching strategies to better characterize the effects of averaging on both rs-fMRI and sMRI spatial patterns.

#### 3.3.2. Analysis of Behavioral Variables with Constrained tIVA

In constrained IVA, a primary objective is to enhance the separability of sources, which is achieved when the applied constraints are as independent as possible. However, in constrained tIVA, the cognitive measures used as constraints often assess similar or overlapping constructs. To better capture the distinct contribution of each cognitive score, the constraints were therefore applied individually in separate runs rather than simultaneously.

For each cognitive score, two-sample *t*-tests were performed on the corresponding profiles to evaluate group differences between healthy controls and patients. The resulting *p*-values were corrected for multiple comparisons using the FDR correction, and the components associated with the significant profiles were examined to interpret the differences across tasks under each constraint.

**Figure 4:**
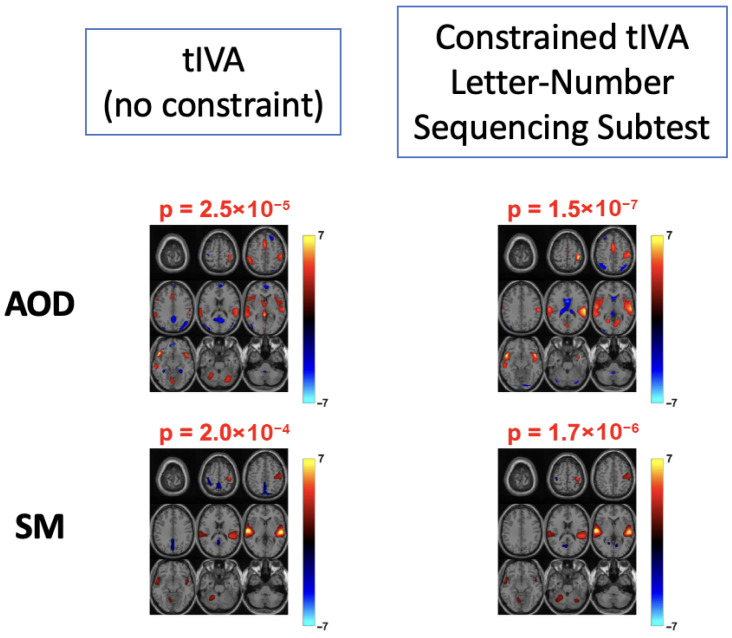
Comparison of components obtained from tIVA and constrained tIVA (with the constraint of letter-number sequencing subtest). It is observed that with the addition of constraints, the activations in this component become more pronounced in task related networks. In addition, the *p*-value corresponding to this component is lower, indicating a stronger group level difference.

To assess the role of constraints in driving behavioral alignment and group differences, we performed a comparison between constrained tIVA and unconstrained tIVA. For the unconstrained model, components were matched to the constrained components using spatial correlation, and the corresponding group statistics were evaluated. Across behavioral variables, unconstrained tIVA components exhibited weaker spatial activations within task relevant regions and reduced group level statistical significance compared with their constrained counterparts. Figure 4 illustrates a representative example for the Letter–Number Sequencing subtest, where constrained tIVA yields more pronounced activations in task-relevant auditory regions and a substantially lower group-difference *p*-value relative to the best-matched unconstrained component. These results indicate that imposing behavioral constraints plays a critical role in guiding component alignment toward behaviorally meaningful brain patterns and in enhancing sensitivity to group effects.

**Figure 5:**
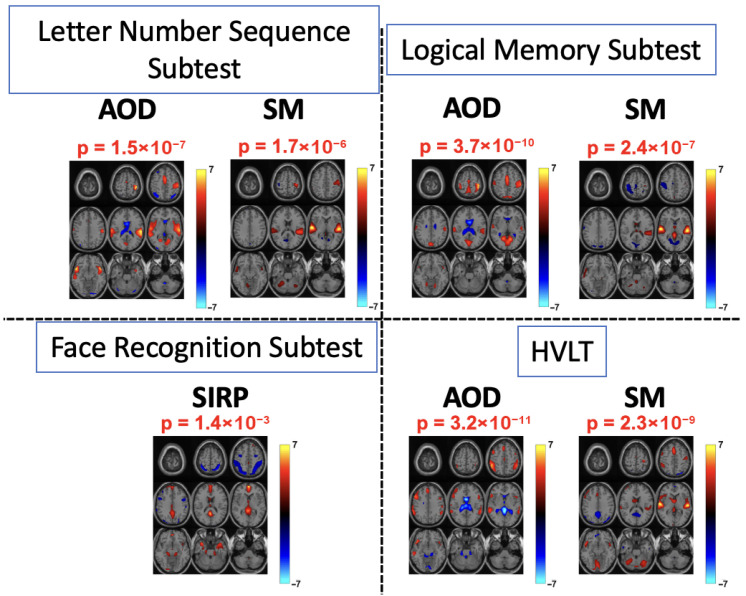
Spatial components corresponding to each behavioral variable constraint. For all four behavioral variables, these components show significant group differences in key networks, including the auditory network, sensorimotor network, and angular gyrus. For the face recognition subtest, a measure of visual memory, we observed significant activation differences between groups in visual regions, particularly within the SIRP task, which is also visually based. Similarly, we found more interpretable and significant activations for the letter–number sequencing subtest, logical memory subtest, and HVLT under the AOD and SM tasks. The *p*-values of the estimated components were highly significant across all four behavioral constraints, consistent with the group differences observed in the corresponding behavioral variables.

Figure 5 demonstrates the components obtained from tIVA by constraining the profile information. For the Letter–Number Sequencing subtest, the components estimated by constrained transposed IVA revealed significant group differences for the AOD and SM tasks, with activations primarily observed in the auditory and sensorimotor networks. In schizophrenia, sensory-level contributions to working memory are often impaired, reflecting disrupted integration between sensory networks and frontoparietal control networks [48,49]. During the AOD and SM tasks, the auditory network maintains and updates stimulus representations essential for working memory, while the activation of sensorimotor regions likely reflects the response preparation. The observed group differences within these networks are consistent with such deficits in working memory and align with previous findings using the same behavioral variable [12].

As a measure of visual memory, the face recognition subtest, was used to assess visual memory, and the most prominent task-related activations were observed during the SIRP task, consistent with its visual nature. The largest activation difference was found in the angular gyrus, a key region for memory retrieval. As part of the DMN, the angular gyrus is typically deactivated during cognitive tasks such as SIRP. However, schizophrenia patients often exhibit an impaired ability to suppress DMN activity [50,51], which explains the elevated activation observed in the angular gyrus for this group. In terms of the statistical significance, compared with other behavioral variables, the *p*-value was higher, indicating less significant difference. This was expected, since the *p*-value associated with this behavioral variable was higher than for other measures, reflecting a smaller group difference. In addition, it has been noted in previous work that visual regions are often not assessed in studies of higher cognitive dysfunction for schizophrenia patients [49].

The logical memory subtest and the Hopkins verbal learning test (HVLT) were used to evaluate verbal memory and learning. Within the SM task, significant activations were observed in the auditory network for these behavioral measures. Logical memory tasks involve both encoding and recall of auditory stimuli, and sensory-level disruptions in schizophrenia likely contribute to the observed group differences in auditory network activations. For the AOD task, activations were primarily observed in sensorimotor and bilateral frontoparietal regions. The frontoparietal network, a core component of the central executive network, is responsible for short-term memory retrieval and language-based processing [52], and is frequently implicated in memory deficits in schizophrenia [31]. These functional differences explain the significant activation differences detected in the AOD task.

Table 2 provides a compact summary of the findings across all four behavioral constraints. In addition to the *p*-values obtained from the statistical analysis, which indicate significant group differences, the corresponding effect sizes (Cohen’s d) are also reported. The calculated effect sizes were all above 0.5 and reached values as high as 0.8 for some behavioral variables, further supporting the practical significance of the observed group differences.

By applying constrained tIVA, we were able to directly identify source components associated with each behavioral variable, eliminating the need for secondary analyses (e.g., correlation calculations) that would typically be required under a standard IVA framework. Furthermore, since tIVA performs dimensionality reduction at the voxel level rather than at the subject level (V≫K), the resulting datasets are substantially smaller in dimension. This reduction not only simplifies the computational process but also leads to a significant decrease in computation time.

## 4. Summary

In this study, we investigated guided IVA approaches for multi-task fMRI data using both standard and transposed formulations. Constrained IVA was first applied with two sets of references, rs-fMRI and sMRI, to evaluate their effectiveness in guiding source separation. Our results demonstrate that sMRI references provide a better match for task-based fMRI feature data compared with rs-fMRI references. This improvement comes from the closer modality alignment between sMRI and task fMRI data, as well as inherent differences between resting-state and task-related activations. As a part of future work, task fMRI data without regression to task response can be analyzed to further investigate whether sMRI references remain a better match under this modality. Using sMRI references, we successfully identified significant components within task-relevant networks, such as the sensorimotor network, with higher correlations to the reference components relative to rs-fMRI guided results.

Under the constrained tIVA framework, we further examined the associations between brain activations and behavioral variables in a novel and efficient manner. Constrained tIVA enabled the identification of activation patterns related to each cognitive measure across multiple tasks without requiring secondary analyses. Significant group differences were observed for all four behavioral variables, primarily involving the sensory motor, auditory, default mode, frontoparietal, and angular gyrus regions. These findings highlight functional deficits in working, verbal, and visual memory among schizophrenia patients, consistent with previous clinical studies. Overall, our results demonstrate the utility of constrained IVA and tIVA frameworks in revealing meaningful functional and behavioral associations in multi-task fMRI data.

## Figures and Tables

**Table 1:** Correlation between the estimated source components and references (values are bold when the correlation value with sMRI reference is higher).

Task	Reference	SC	AU	SM	VI	CC	DM	CB
AOD	sMRI	**0.6763**	0.4983	**0.5011**	**0.6651**	0.3824	**0.6117**	**0.7304**
AOD	fMRI	0.4690	0.5053	0.4651	0.5741	0.4690	0.6043	0.6608
SIRP	sMRI	**0.6513**	0.5060	**0.5162**	**0.6709**	0.4147	**0.5928**	**0.7120**
SIRP	fMRI	0.4510	0.5440	0.4572	0.5935	0.4627	0.5844	0.6748
SM	sMRI	**0.6845**	**0.5060**	**0.5152**	**0.6764**	0.4250	**0.6026**	**0.7278**
SM	fMRI	0.4389	0.4444	0.4559	0.5847	0.4720	0.5836	0.6793

**Table 2:** Summary table showing *p*-values, Cohen’s d effect sizes, and the networks in which activations were observed for each task and behavioral variable. (AUD: auditory, SM: sensorimotor, AG: angular gyrus, DM: default mode, FP: frontoparietal).

Tasks	Letter Number Sequencing *Measures Working Memory*			Logical Memory *Measures Verbal Memory and Learning*			Face Recognition *Measure Visual Memory*			HVLT *Measure Verbal Memory and Learning*		
	p	d	Networks	p	d	Networks	p	d	Networks	p	d	Networks
AOD	$1.5\times 10^{-7}$	0.69	AUD, SM	$3.7\times 10^{-10}$	0.81	SM	–	–	–	$3.2\times 10^{-11}$	0.84	FP
SIRP	–	–	–	–	–	–	$1.4\times 10^{-3}$	0.53	AG, DM	–	–	–
SM	$1.7\times 10^{-6}$	0.64	AUD	$2.4\times 10^{-7}$	0.69	AU	–	–	–	$2.3\times 10^{-9}$	0.77	AU

## Data Availability

The datasets used in this study are publicly available at https://coins.trendscenter.org/, accessed on 15 October 2025.

## Abbreviations

The following abbreviations are used in this manuscript:

fMRI	Functional magnetic resonance imaging
IVA	Independent vector analysis
ICA	Independent component analysis
tIVA	Transposed independent vector analysis
sMRI	Structural magnetic resonance imaging
rs-fMRI	Resting-state functional magnetic resonance imaging
AOD	Auditory Oddball
SIRP	Sternberg Item Recognition Paradigm
SM	Sensory Motor
SCV	Source Component Vector
tf-civa	Threshold-free constrained independent vector analysis
WAIS-III	Wechsler Adult Intelligence Scale-Third Edition
WMS-III	Wechsler Memory Scale-Third Edition
HVLT	Hopkins Verbal Learning Test–Revised
FDR	False Discovery Rate
Cross-joint-ISI	Cross-joint inter-symbol interference
SC	Subcortical
AU	Auditory
VI	Visual
CC	Cognitive control
DM	Default mode
CB	Cerebellar

## References

[1] Lahat D., Adalı T., Jutten C. (2015). Multimodal Data Fusion: An Overview of Methods, Challenges, and Prospects. Proc. IEEE.

[2] Acar E., Schenker C., Levin-Schwartz Y., Calhoun V.D., Adalı T. (2019). Unraveling Diagnostic Biomarkers of Schizophrenia Through Structure-Revealing Fusion of Multi-Modal Neuroimaging Data. Front. Neurosci..

[3] McGuire P.K., Matsumoto K. (2004). Functional Neuroimaging in Mental Disorders. World Psychiatry.

[4] Itkyal V., Iraji A., Jensen K.M., LaGrow T.J., Duda M., Turner J.A., Liu J., Wu L., Du Y., Fries J. (2025). Evidence for White Matter Intrinsic Connectivity Networks at Rest and During a Task: A Large-Scale Study and Templates. Netw. Neurosci..

[5] Calhoun V.D., Adalı T., Kiehl K.A., Astur R., Pekar J.J., Pearlson G.D. (2006). A Method for Multitask fMRI Data Fusion Applied to Schizophrenia. Hum. Brain Mapp..

[6] Adalı T., Anderson M., Fu G. (2014). Diversity in Independent Component and Vector Analyses: Identifiability, Algorithms, and Applications in Medical Imaging. IEEE Signal Process. Mag..

[7] Adalı T., Akhonda M.A.B.S., Calhoun V.D. (2019). ICA and IVA for Data Fusion: An Overview and a New Approach Based on Disjoint Subspaces. IEEE Sens. Lett..

[8] Kim T., Attias H.T., Lee S.-Y., Lee T.-W. (2007). Blind Source Separation Exploiting Higher-Order Frequency Dependencies. IEEE Trans. Audio Speech Lang. Process..

[9] Anderson M., Adalı T., Li X.-L. (2012). Joint Blind Source Separation with Multivariate Gaussian Model: Algorithms and Performance Analysis. IEEE Trans. Signal Process..

[10] Lehmann I., Hasija T., Gabrielson B., Akhonda M.A.B.S., Calhoun V.D., Adalı T. Multi-Task fMRI Data Fusion Using IVA and PARAFAC2. Proceedings of the ICASSP 2022–2022 IEEE International Conference on Acoustics, Speech and Signal Processing (ICASSP).

[11] Lehmann I., Hasija T., Gabrielson B., Akhonda M.A.B.S., Calhoun V.D., Adalı T. (2024). Identifying the Relationship Structure Among Multiple Datasets Using Independent Vector Analysis: Application to Multi-Task fMRI Data. IEEE Access.

[12] Akhonda M.A.B.S., Levin-Schwartz Y., Calhoun V.D., Adalı T. (2022). Association of Neuroimaging Data with Behavioral Variables: A Class of Multivariate Methods and Their Comparison Using Multi-Task fMRI Data. Sensors.

[13] Adalı T., Levin-Schwartz Y., Calhoun V.D. (2015). Multimodal Data Fusion Using Source Separation: Two Effective Models Based on ICA and IVA and Their Properties. Proc. IEEE.

[14] Salman M.S., Levin-Schwartz Y., Adalı T., Calhoun V.D. (2019). Group ICA for Identifying Biomarkers in Schizophrenia: ‘Adaptive’ Networks via Spatially Constrained ICA Show More Sensitivity to Group Differences than Spatio-Temporal Regression. Neuroimage Clin..

[15] Bhinge S., Levin-Schwartz Y., Adalı T., Calhoun V.D. (2019). Extraction of Time-Varying Spatiotemporal Networks Using Parameter-Tuned Constrained IVA. IEEE Trans. Med. Imag..

[16] Vu T., Laport F., Yang H., Calhoun V.D., Adalı T. (2024). Constrained Independent Vector Analysis with Reference for Multi-Subject fMRI Analysis. IEEE Trans. Biomed. Eng..

[17] Kumbasar E.E., Yang H., Vu T., Calhoun V.D., Adalı T. Fusion of Multitask fMRI Data with Constrained Independent Vector Analysis. Proceedings of the 59th Annual Conference on Information Sciences and Systems (CISS).

[18] Zhang S., Li X., Lv J., Jiang X., Guo L., Liu T. (2016). Characterizing and Differentiating Task-Based and Resting State fMRI Signals via Two-Stage Sparse Representations. Brain Imaging Behav..

[19] Calhoun V.D., Allen E. (2013). Extracting Intrinsic Functional Networks with Feature-Based Group Independent Component Analysis. Psychometrika.

[20] Ellis D.G., Aizenberg M.R. (2022). Structural Brain Imaging Predicts Individual-Level Task Activation Maps Using Deep Learning. Front. Neuroimaging.

[21] Luo N., Sui J., Abrol A., Chen J., Turner J.A., Damaraju E., Fu Z., Fan L., Lin D., Zhuo C. (2020). Structural Brain Architectures Match Intrinsic Functional Networks and Vary across Domains: A Study from 15,000+ Individuals. Cereb. Cortex.

[22] Fu Z., Batta I., Wu L., Abrol A., Agcaoglu O., Salman M.S., Du Y., Iraji A., Shultz S., Sui J. (2024). Searching Reproducible Brain Features Using NeuroMark: Templates for Different Age Populations and Imaging Modalities. NeuroImage.

[23] Bigler E.D., Sanders K.M. (2019). Neuroimaging and Neuropsychology. Physician’s Field Guide to Neuropsychology: Collaboration Through Case Example.

[24] Meng X., Jiang R., Lin D., Bustillo J., Jones T., Chen J., Yu Q., Du Y., Zhang Y., Jiang T. (2017). Predicting Individualized Clinical Measures by a Generalized Prediction Framework and Multimodal Fusion of MRI Data. NeuroImage.

[25] Sui J., Jiang R., Bustillo J., Calhoun V.D. (2020). Neuroimaging-Based Individualized Prediction of Cognition and Behavior for Mental Disorders and Health: Methods and Promises. Biol. Psychiatry.

[26] Young L., Koenigs M. (2007). Investigating Emotion in Moral Cognition: A Review of Evidence from Functional Neuroimaging and Neuropsychology. Br. Med. Bull..

[27] Gollub R.L., Shoemaker J.M., King M.D., White T., Ehrlich S., Sponheim S.R., Van Erp T.G.M., Turner J.A., Potkin S.G., Mathalon D.H. (2013). The MCIC Collection: A Shared Repository of Multi-Modal, Multi-Site Brain Image Data from a Clinical Investigation of Schizophrenia. Neuroinformatics.

[28] Wechsler D. (1997). WAIS-III Administration and Scoring Manual.

[29] Wechsler D. (1997). WAIS-3, WMS-3: Wechsler Adult Intelligence Scale, Wechsler Memory Scale: Technical Manual.

[30] Brandt J. (1991). The Hopkins Verbal Learning Test: Development of a New Memory Test with Six Equivalent Forms. Clin. Neuropsychol..

[31] Qi S., Calhoun V.D., van Erp T.G.M., Bustillo J., Damaraju E., Turner J.A., Du Y., Yang J., Chen J., Yu Q. (2018). Multimodal Fusion with Reference: Searching for Joint Neuromarkers of Working Memory Deficits in Schizophrenia. IEEE Trans. Med. Imaging.

[32] Glahn D.C., Ragland J.D., Abramoff A., Barrett J., Laird A.R., Bearden C.E., Velligan D.I. (2005). Beyond Hypofrontality: A Quantitative Meta-Analysis of Functional Neuroimaging Studies of Working Memory in Schizophrenia. Hum. Brain Mapp..

[33] Benjamini Y., Yakuteli D. (2005). False Discovery Rate–Adjusted Multiple Confidence Intervals for Selected Parameters. J. Am. Stat. Assoc..

[34] Cohen J. (1988). Statistical Power Analysis for the Behavioral Sciences.

[35] Long Q., Jia C., Boukouvalas Z., Gabrielson B., Emge D., Adalı T. Consistent Run Selection for Independent Component Analysis: Application to fMRI Analysis. Proceedings of the 2018 IEEE International Conference on Acoustics, Speech and Signal Processing (ICASSP).

[36] Adali T., Calhoun V.D. (2022). Reproducibility and Replicability in Neuroimaging Data Analysis. Curr. Opin. Neurol..

[37] Members and Collaborators of the Welcome Centre for Human Neuroimaging (2020). Statistical Parametric Mapping Toolbox: SPM12.

[38] Michael A.M., Baum S.A., Fries J.F., Ho B.C., Pierson R.K., Andreasen N.C., Calhoun V.D. (2009). A method to fuse fMRI tasks through spatial correlations: Applied to schizophrenia. Hum. Brain Mapp..

[39] Kiehl K.A., Liddle P.F. (2001). An Event-Related Functional Magnetic Resonance Imaging Study of an Auditory Oddball Task in Schizophrenia. Schizophr. Res..

[40] Du Y., Fu Z., Sui J., Gao S., Xing Y., Lin D., Salman M., Abrol A., Rahaman M.A., Chen J. (2020). NeuroMark: An Automated and Adaptive ICA Based Pipeline to Identify Reproducible fMRI Markers of Brain Disorders. NeuroImage Clin..

[41] Jimenez A.M., Riedel P., Lee J., Reavis E.A., Green M.F. (2019). Linking Resting-State Networks and Social Cognition in Schizophrenia and Bipolar Disorder. Hum. Brain Mapp..

[42] Lottman K.K., Gawne T.J., Kraguljac N.V., Killen J.F., Reid M.A., Lahti A.C. (2019). Examining Resting-State Functional Connectivity in First-Episode Schizophrenia with 7T fMRI and MEG. NeuroImage Clin..

[43] Du W., Calhoun V., Li H., Ma S., Eichele T., Kiehl K., Pearlson G., Adalı T. (2012). High Classification Accuracy for Schizophrenia with Rest and Task fMRI Data. Front. Hum. Neurosci..

[44] Manoach D.S., Gollub R.L., Benson E.S., Searl M.M., Goff D.C., Halpern E., Saper C.B., Rauch S.L. (2000). Schizophrenic Subjects Show Aberrant fMRI Activation of Dorsolateral Prefrontal Cortex and Basal Ganglia During Working Memory Performance. Biol. Psychiatry.

[45] Hu M., Zong X., Mann J.J., Zheng J., Liao Y., Li Z., He Y., Chen X., Tang J. (2017). A Review of the Functional and Anatomical Default Mode Network in Schizophrenia. Neurosci. Bull..

[46] Kim D., Manoach D., Mathalon D., Turner J., Mannell M., Brown G., Ford J., Gollub R., White T., Wible C. (2009). Dysregulation of Working Memory and Default-Mode Networks in Schizophrenia During a Sternberg Item Recognition Paradigm. Hum. Brain Mapp..

[47] Abramian D., Sidén P., Knutsson H., Villani M., Eklund A. Anatomically Informed Bayesian Spatial Priors for fMRI Analysis. Proceedings of the 2020 IEEE 17th International Symposium on Biomedical Imaging (ISBI).

[48] D’Esposito M., Postle B.R. (2015). The Cognitive Neuroscience of Working Memory. Annu. Rev. Psychol..

[49] Javitt D.C., Freedman R. (2014). Sensory Processing Dysfunction in the Personal Experience and Neuronal Machinery of Schizophrenia. Am. J. Psychiatry.

[50] Whitfield-Gabrieli S., Thermenos H.W., Milanovic S.K., Tsuang M.T., Faraone S.V., McCarley R.W., Shenton M.E., Green A.I., LaViolette P., Wojcik J.R. (2009). Hyperactivity and Hyperconnectivity of the Default Network in Schizophrenia and in First-Degree Relatives of Persons with Schizophrenia. Proc. Natl. Acad. Sci. USA.

[51] Hahn B., Harvey P.O., Green M.F., Keshavan M., Wynn J.K., Horan W.P., Gold J.M. (2016). Hyperdeactivation of the Default Mode Network in People with Schizophrenia When Focusing Attention in Space. Schizophr. Bull..

[52] Chan Y.-C., Lavallee J.P. (2015). Temporo-Parietal and Fronto-Parietal Lobe Contributions to Theory of Mind and Executive Control: An fMRI Study of Verbal Jokes. Front. Psychol..

